# Analysis of barriers and facilitators to early hearing detection and intervention in KwaZulu-Natal, South Africa

**DOI:** 10.4102/sajcd.v69i1.839

**Published:** 2022-01-31

**Authors:** Naedene Naidoo, Nasim B. Khan

**Affiliations:** 1 Discipline of Audiology, School of Health Sciences, University of KwaZulu-Natal, Westville Campus, Durban, South Africa

**Keywords:** EHDI, SWOT analysis, barriers, facilitators, South Africa

## Abstract

**Background:**

There is slow progress in early hearing detection and intervention (EHDI) services within South Africa. Audiologists are EHDI gatekeepers and can provide valuable insights into the barriers and facilitators that can progressively move EHDI towards best practice in South Africa.

**Objectives:**

The study aimed to determine the barriers and facilitators to EHDI in KwaZulu-Natal as reported by audiologists/speech therapists and audiologists (A/STAs).

**Method:**

A descriptive qualitative approach was used. Telephonic interviews were conducted with 12 A/STAs working in public and private healthcare facilities, using the strengths, weaknesses, opportunities, threats (SWOT) conceptual framework. Data was analysed using thematic analysis in conjunction with NVivo software.

**Results:**

One of the main barriers perceived by A/STAs, affecting EHDI was the lack of resources in healthcare facilities. The participants indicated that although there was a guideline in place to guide practice, it may be more suited to an urban area versus a rural area. Poor knowledge and awareness of EHDI was also identified as a barrier. Information provided from A/STAs at grassroots level, in the various provinces, may benefit in developing a more contextually relevant and practical guideline. Facilitators included; development of task teams specifically for EHDI programmes, creation of improved communication networks for collaboration and communication, training of healthcare professionals and improving data management systems.

**Conclusion:**

Strategies such as an increase in resources, further education and training, development of contextually relevant, culturally, and linguistically diverse practices and protocols need to be in place to improve EHDI implementation. Further research, clinical implications and limitations are provided emanating from the study.

## Introduction

Hearing loss is seen as a silent epidemic because of its invisible nature, as many clinical examinations fail to identify it (Petersen & Ramma, 2015) and is more predominant in neonates compared to other disorders routinely screened for (Imam, El-Farrash, Taha, & Bishoy, 2013). Undetected hearing loss in a newborn can result in devastating long-term consequences such as emotional disturbance, communication delays, cognitive deficits, and social-emotional problems leading to educational barriers, career limitations, and employment difficulties (Bezuidenhout, Khoza-Shangase, De Maayer, & Strehlau, 2018; Olusanya, 2008).

In South Africa, approximately 6357 children annually are born with a permanent hearing loss or develop it at an early age, with the majority being born in the public healthcare sector (Teixeira & Joubert, 2014). The prevalence of infants born with a permanent hearing loss in the public sector in South Africa is 3–6 per 1000 births (Khoza-Shangase, Kanji, Petrocchi-Bartal, & Farr, 2017; Michal & Khoza-Shangase, 2014).

The early hearing detection and intervention (EHDI) guidelines were released by the Health Professions Council of South Africa (HPCSA) (HPCSA, 2007, 2018). EHDI programmes are the proposed standard of care for newborns and infants presenting with a hearing loss, enabling them to develop to their full potential (HPCSA, 2018). Universal newborn hearing screening (UNHS), one of the key EHDI imperatives, is the standard practice internationally which is mainly limited to the developed world, yet the majority of hearing-impaired children are living in developing countries (Harbinson & Khoza-Shangase, 2015).

Research evidence has suggested that UNHS may not be attainable in South Africa, particularly in the public healthcare sector, because of insufficient resources, manpower, and demand whereby more than 80% of the population access public healthcare services (Kanji, 2016; Khoza-Shangase et al., 2017). Furthermore, the public and private sectors also have other challenges including cost, poor infrastructure, and limited hearing screening programmes, thus affecting the implementation of newborn hearing screening (NHS) across low-and-middle-income countries (Krishnan & Donaldson, 2013). This is further exacerbated, as additional burdens such as poverty or life-threatening conditions, that is tuberculosis (TB) and human immunodeficiency virus and/or acquired immunodeficiency syndrome (HIV and/or AIDS), are viewed as an urgent priority, whilst hearing loss may be viewed as less urgent (Petrocchi-Bartal & Khoza-Shangase, 2016). Moreover, the COVID-19 pandemic which has taken the world by surprise has exacerbated the challenges to conducting NHS (Yoshinaga-Itano, 2020). NHS services may be operational, but family concerns can be present when taking babies for screening or diagnostic assessments during the crisis, so reassurances and counselling are essential to make sure babies are provided with timely services (Yoshinaga-Itano, 2020).

Few healthcare facilities in South Africa offer NHS, resulting in late diagnosis and intervention among these children (Petersen & Ramma, 2015; Swanepoel, 2006; Swanepoel, Delport, & Swart, 2004). Improvement in the initial age of screening was noted from a retrospective study conducted in Johannesburg, which revealed that the median age for initial hearing screening was 11 months (Opperman & Kanji, 2015). Unfortunately, this still does not meet the HPCSA EHDI stipulated guidelines, which states hearing screening by 1 month and no later than 6 weeks and intervention by 6 months and no later than 8 months (HPCSA, 2007, 2018). However, this is a positive finding which emphasises that with continued implementation, the guidelines can be met, as compared to previous studies that have shown later identification (Van der Spuy & Pottas, 2008; Butler et al., 2013; Khoza-Shangase & Michal, 2014; Swanepoel et al., 2013; Störbeck & Young, 2016).

There seems to be a limited collaboration between stakeholders and other professionals such as nurses, early interventionists, and otolaryngologists in developing the EHDI guideline, which is important for successful implementation of UNHS. Educational level, family income, parental perception, and social factors are other barriers impacting timely screening, follow-up, and management (Yun et al., 2017). It is therefore important to gather information about parental or caregiver perspectives on NHS to ensure appropriate advocacy and interventions (Jatto, Ogunkeyede, Adeyemo, Adeagbo, & Saiki, 2018). Furthermore, NHS programmes are not mandatory in South Africa (Bezuidenhout et al., 2021), thus necessitating parents being aware and knowledgeable of the risk factors for infant hearing loss (Govender & Khan, 2017).

The stigma that is associated with deafness can be a barrier preventing individuals from accessing rehabilitative services (Das, Seepana, & Bakshi, 2020). Further challenges identified from Merugumala, Pothula and Cooper’s (2017) study related to cultural, educational, transportation, and financial barriers affecting the access to services, especially those in rural areas and from lower socioeconomic statuses. Intervention services are usually provided at health facility level (HPCSA, 2018). Thus, access to support, assessment and intervention may be challenging for the vulnerable population or families (Samuels, Slemming, & Balton, 2012). Cultural beliefs may be associated with hearing loss, thus healthcare professionals should demonstrate cultural competence when providing services, in countries that are culturally and linguistically diverse, like South Africa (Govender & Khan, 2017). Parents of a child who is deaf, may spend large sums of money in the beginning, visiting orthodox medical practitioners thereafter traditional healers before receiving rehabilitative intervention (Rajagopalan, Selvarajan, Rajendran, & Ninan, 2014). These attitudes may arise from indigenous traditions attributed to the stigma of hearing loss and deafness, leading to resistance to hearing aids and sign language (Rajagopalan et al., 2014). South Africa is a multicultural society and the context and the manner in which information is provided to parents regarding the importance of hearing screening is imperative (Moodley & Störbeck, 2012) as well as follow-up services.

The majority of studies that have been published focus on the implementation and the challenges or barriers to EHDI services (Khoza-Shangase, Barrett, & Jonosky, 2010; Petrocchi-Bartal & Khoza-Shangase, 2014; Theunissen & Swanepoel, 2008). However, the strengths and achievements should also be identified for ensuring best practice and standard of care in EHDI service delivery. Thus, evaluation of the feasibility of the HPCSA EHDI guidelines and practices, to determine the barriers and facilitators is essential within the South African context (Petrocchi-Bartal & Khoza-Shangase, 2014), specifically in KwaZulu-Natal (KZN).

This study aimed to explore the barriers and facilitators to EHDI in KZN, as reported by audiologists/speech therapists, and audiologists (A/STAs). The objectives of the study were to (1) describe the weaknesses and threats (barriers), and (2) describe the strengths and opportunities (facilitators) identified by A/STAs to EHDI.

## Methods and materials

### Study design

An exploratory-descriptive research design was used in this study with qualitative methods of analysis. Telephonic interviews were conducted with all the participants.

### Study population and sample

Purposive, convenience sampling enabled the researcher to choose 12 A/STAs conveniently located in public healthcare facilities and private practices, in KZN. The participants had to be registered with the HPCSA and employed in healthcare facilities (private or public) within KZN. Therapists that were dually registered working in KZN were also included and individuals of different races, ages, and gender were included to ensure diversity.

The pilot study was conducted with two A/STAs, who were not a part of the main study. The results from the pilot study indicated that the data collection method and interview time were appropriate. No concerns were reported regarding the understanding of the questions. However, one participant indicated that some of the questions were too long and required multiple aspects. Another concern reported by one participant was that there should be questions about the diagnostic and intervention aspects of EHDI, and not only on screening. Therefore, adjustments were made to the content of the telephonic interview schedule, questions were separated and more probing questions were added.

### Data collection tool

Telephonic interviews were used to collect detailed information, in a semi-structured session through the use of general guideline questions (Carey & Asbury, 2016). It took between 45 min and 60 min to conduct an interview, and it was audio-recorded. The telephonic interview schedule (Appendix 1) comprised of 8 main open-ended questions, which targeted the areas related to the guidelines, NHS, initial age of screening, screening protocols and platforms, age of diagnosis and intervention, loss to follow-up, and data management, which allowed the researcher to gather rich contextually relevant, informal and free-flowing information, enabling the participants to respond in any way they chose (Brace, 2013; Magnusson & Marecek, 2015).

Probe questions were further adapted from White and Blaiser’s (2011) questionnaire and all principles mentioned in the HPCSA EHDI 2007 and 2018 guidelines were included.

**FIGURE 1:**
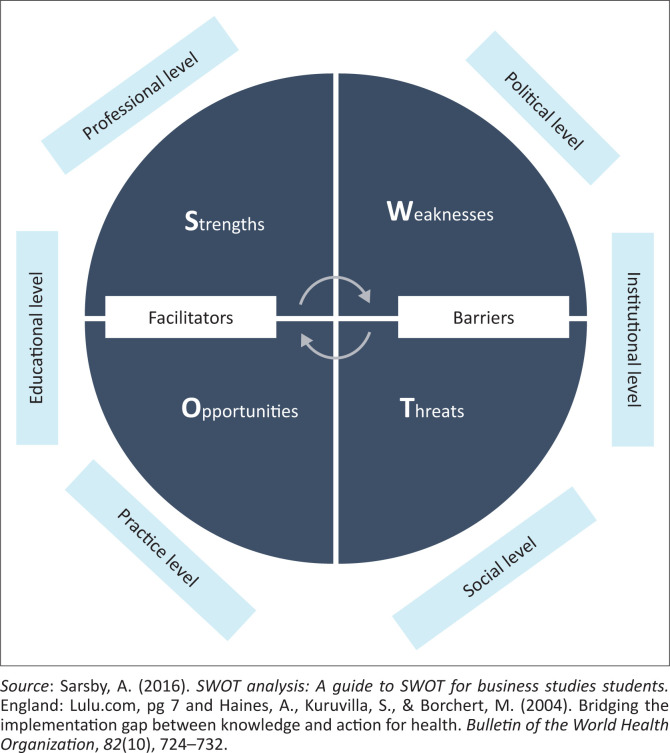
Conceptual strengths, weaknesses, opportunities, threats framework adapted for this study.

A strengths, weaknesses, opportunities, threats (SWOT) conceptual framework (see Figure 1) was used to guide the telephonic interviews. The conceptual framework was used to learn about the experiences from A/STAs to enable the researcher to cultivate their perspective and knowledge (Ravitch & Riggan, 2012, 2016). The SWOT analysis contrasts and compares weaknesses, strengths, threats, and opportunities to a set of criteria (Silva et al., 2014). The SWOT is a qualitative research tool that observes external factors (threats and opportunities) and internal factors (weaknesses and strengths) (Silva et al., 2014). A comprehensive analysis allows one to capitalise on the advantages or strengths and provides the foresight to detect looming threats to prepare (Sarsby, 2016; Silva et al., 2014).

Furthermore, aspects from EHDI guideline generation through to clinical application incorporating various levels, that is professional, political, social, institutional, practice, and educational levels that could affect EHDI service provision was adapted from Haines, Kuruvilla and Borchert (2004). Professional level factors are the standards of practice, policies and guidelines, and the appropriateness, practicality, and feasibility of these towards rendering EHDI services in the South African context. Political level areas include the governmental departments of health or education who play a big role in the decision-making process. Institutional level factors include human resources equipment, type, structure, and area of healthcare facilities. Practice level areas include the protocols in healthcare facilities, healthcare professionals’ practices, and settings of practice. Social level factors include people’s beliefs about the health system and their health-seeking behaviours. Educational level areas include knowledge, information, and training about health services by healthcare professionals, parents or caregivers, and the government.

### Study procedure

Identified participants were contacted through phone calls, and the informed consent letter was emailed to them. Once confirmation was received, details of the interview, such as time and date were scheduled. The main study consisted of 12 telephonic interviews as the saturation point was reached and no new information was being obtained. The data was then transcribed and member checking was conducted. Copies of the transcribed interviews were emailed to the participants to check for their accuracy (Kiyimba, Lester, & O’Reilly, 2018) and ensure the credibility and trustworthiness of the data. The participants were given 3 to 5 days to check and email the transcribed interviews back to the researcher, with a total of six participants (50%) emailing back and no changes being made to the transcribed interviews.

### Data analysis

A statistician was contacted to aid with the data analysis. The data for each question was inputted on an excel spreadsheet, cleaned, and then imported to NVivo for further analysis. NVivo is a qualitative data analysis programme that makes the analysis of qualitative data easier and efficient by coding the data (Opie & Brown, 2019). NVivo was used as the sole coding method for the first round of data analysis and is reported to be the best method for small-scale studies (Saldana, 2012), with the second part involving inductive thematic analysis, whereby categories were used to label similar coded data (Saldana, 2012; Willing & Rogers, 2017).

### Ethical considerations

The proposed research study was sent to the University of KZN Humanities and Social Science Research Ethics Committee to obtain the ethical approval (reference number: HSSREC/00001003/2020) to conduct the study. The Helsinki declaration was used to ensure that human participants’ rights were protected and treated with respect (Gallin, Ognibene, & Johnson, 2017). All data collected was available only to the researcher and supervisor. Participant anonymity and confidentiality were maintained throughout the study. An informed consent letter was provided to the participants once they were identified, as consent is voluntary and participants should be aware that they can withdraw at any time during the study (Bhattacherjee, 2012; Crowther & Lauesen, 2017). The data will be stored for 5 years and will be disposed of in January 2025, through shredding.

## Results and discussion

Most participants were female, between the ages of 21 and 30 years, having a bachelor’s degree, employed in public healthcare facilities, in an urban area, and within the eThekwini district.

The results and discussion are present according to the five main themes. These include: (1) EHDI guideline improvements; (2) investment in resources and infrastructure for EHDI; (3) facilitating and strengthening intersectoral collaboration; (4) evaluating protocols and procedures for screening and managing follow-up; and (5) engaging, understanding, and supporting caregivers or families

### Early hearing detection and intervention guideline improvements

The first theme related to EHDI guideline improvements and included the aspects linked to guideline development, implementation, and review. The feedback received in relation to this theme is summarised in Table 1.

**TABLE 1:** Strengths, weaknesses, opportunities, and threats for theme 1: Early hearing detection and intervention guideline improvements.

Variable	Subthemes: Guideline development; guideline implementation and guideline review.
Strengths	Guideline exists to direct professional practice Guideline is evidence-based Contextual adjustments are evident Widespread support for EHDI
Weaknesses	Lack of consultation with people on the ground Lack of a national database Limited recording and reporting of EHDI Variability in data capturing The guideline is not reviewed and updated regularly
Opportunities	The guideline should be adapted per district Invest in the creation of a national database
Threats	The diverse context that audiologists work in can present challenges to EHDI Too much emphasis on initial screening

EHDI, early hearing detection and intervention.

All of the participants reported that at a professional and institutional level it was beneficial that there is an evidence-based guideline with contextual adjustments being evident to direct practice, but felt that it was more suited towards private compared to public healthcare. The participants believed that the people developing the guidelines or policies are not always the individuals working at ground level, which was one of the weaknesses identified as stated in Table 1. Furthermore, the participants believed that the guideline should have been revised or reviewed more regularly and not after approximately 10 years. A major weakness reported was that there is a lack of EHDI statistical data for South Africa, as there is no national data management system. However, most participants stated that an online or computer-based data capturing system would not be practical in certain rural areas, because of a lack of resources. An opportunity identified would be to develop a general protocol based on the HPCSA EDHI guidelines, for each area or district to ensure that it was more relevant to specific contexts. A recommendation for data management identified by one participant was the use of a tracking system such as the assistive devices electronic management system (ADEMS):

‘It is a good way in terms of you know going paperless … and … sort of you know… [*getting*] all the information on one database and things like that. I think in terms of the practicality of it … it’s a bit iffy … you could say ….’ (Participant C, female, bachelor’s degree, public sector)

The participants also felt that there is too much emphasis on the initial screening and that there is little information about follow-up and monitoring of hearing status.

EHDI remains an important need in Africa due to the global incidence and prevalence rates of childhood hearing loss (Khoza-Shangase & Kanji, 2021). Studies have identified the impracticalities of implementing first-world models for hearing screening, in low-and-middle-income countries (Swanepoel et al., 2004; Swanepoel, Hugo, & Louw, 2005). Continuously evaluating hearing screening position statements or guidelines, facilitates evidence-based practice and ensures programme implementation (Khoza-Shangase et al., 2017). Evaluation and interrogation of the feasibility of hearing screening programmes by A/STAs, are critical in determining the practicality of the HPCSA EHDI guidelines and associated UNHS benchmarks, within the South African context (Petrocchi-Bartal & Khoza-Shangase, 2014). A top-down approach seems to be used for developing the EHDI guidelines and even though there is some involvement by stakeholders at the ground level, it is limited. An EHDI programme needs to be established by each of the country’s nine provinces, who will then be responsible for improving and maintaining the services (Khoza-Shangase & Kanji, 2021). The position statements of the Joint Committee of Infant Hearing (JCIH), the American Academy of Pediatrics (AAP), and the World Health Organization (WHO) Newborn and Infant Hearing Screening documents were used to guide the development of the EHDI guidelines. However, despite the availability of the guidelines and adaptations to the South African context, there are still gaps in their implementation.

A study conducted in the United States of America (US) revealed that 91.4% of healthcare facilities were compliant in reporting paediatric hearing data to state EHDI programmes (Chung et al., 2017). Unfortunately, in South Africa, one of the challenges is that there is no standardised national data management system in place, which is a huge weakness impacting how the programme is evaluated, monitored, and reviewed. A national database is needed to correlate data uniformly and facilitate communication with screening and intervention services (HPCSA, 2018). According to the guidelines, it has been recommended that for data collection the provincial coordinators develop and circulate an Excel template (HPCSA, 2018), but the completion of an Excel document was reported to not be feasible.

Nevertheless, one needs to be cognisant when implementing an online or electronic system, as challenges include limited staff for data entry and lack of time (Moodley & Störbeck, 2017). According to the study conducted by Joubert and Casoojee (2013) at primary healthcare clinics in Gauteng on nurses performing immunisations, the majority of nurses did not even record screening results on either card or chart (Road-to-Health Chart and City of Johannesburg Child Health Services Blue card). The absence of a proper database or data management system is a threat affecting the monitoring of NHS in South Africa. A proper database will assist in obtaining accurate prevalence rates of infant and newborn hearing loss in South Africa and can provide information regarding the age of hearing screening and diagnosis (HPCSA, 2018).

### Investment in resources and infrastructure for early hearing detection and intervention

The second theme alluded to resource availability and constraints and included the aspects linked to financial, human, and infrastructure resources. The feedback is summarised in Table 2. One of the main threats identified by the participants, at a political level was the lack of funding or limited budget provided to public healthcare facilities. At an institutional level, the participants stated when A/STAs tried to motivate for equipment in public healthcare facilities, they were always told that there is no budget or funds. All of the participants reported that there is a shortage of audiologists, leading to them conducting targeted or high-risk screening programmes at their healthcare facility only and not UNHS even if the equipment was available. Furthermore, they also reported that a large number of individuals access public healthcare services compared to private, impacting time spent per patient. The participants reported that there seems to be a lack of confidence, especially among community service audiologists, in providing diagnostic services to young children:

‘That’s a large factor in why we are failing, even when we have the equipment, we can still be failing because we’re not good at diagnosing children at a young age.’ (Participant F, female, bachelor’s degree, private sector)

**TABLE 2:** Strengths, weaknesses, opportunities, and threats for theme 2: Investment in resources and infrastructure for early hearing detection and intervention.

Variable	Subthemes: Financial resources; human resources and equipment/infrastructure resources.
Strengths	The private sector is well resourced in terms of staff and equipment than the public sector Private better at adhering to guidelines and able to follow-up with patients
Weaknesses	Community service audiologists unaware of protocols for equipment procurement for EHDI Lack of confidence in testing The large caseload in public may lead to prioritisation of babies with risk factors Lack of equipment
Opportunities	Employing more A/STAs Inclusion of training programmes for community service audiologists More advocacy and workshops The use of tele-audiology be explored Provision of equipment Creation of dedicated task teams
Threats	Insufficient funding/capital Shortage of A/STAs thus difficult to run NHS programmes A/STAs not prioritised for equipment or human resources Inability to maintain, repair, and calibrate equipment Some audiology departments do not have senior audiologists to advise or mentor Long waiting lists in public

EHDI, early hearing detection and intervention; A/STAs, audiologists/speech therapists and audiologists; NHS, newborn hearing screening.

A threat described by the participants was that many public healthcare facilities do not have the appropriate equipment to provide the necessary services. Results from the study indicated that it depends heavily on whether the medical or finance manager sees the importance of getting audiology equipment, compared to medical equipment for doctors. A weakness as stated in Table 2, was the long waiting list or appointment date that is given in public healthcare facilities.

‘[*Y*]ou can’t do screening without follow-up, so if you doing screening you have to go through with your intervention and management thereafter. I will refuse to screen if we can’t … that’s why we don’t do universal screening because we don’t have the manpower and the personnel to follow in terms of intervention, management, hearing aids fittings, etc. You don’t screen and put them on a waiting list … you screen to diagnose and treat and manage them.’ (Participant D, female, bachelor’s degree, public sector)

The translation of policy into practice is greatly influenced because of resource constraints (Khoza-Shangase & Kanji, 2021). The barriers to EHDI in the public sector relate to lack of finance and funding in government and healthcare facilities, affecting the employment of staff and provision of equipment and hearing aids in public healthcare facilities, which is consistently cited in several studies (Khoza-Shangase et al., 2017, Khoza-Shangase & Kanji, 2021; Petrocchi-Bartal & Khoza-Shangase, 2014; Theunissen & Swanepoel, 2008).

Audiologists are crucial for each aspect of the EHDI process (HPCSA, 2007); however, there is a shortage of qualified audiologists in South Africa, compared to the population size, with the most obvious mismatch occurring in the public sector (Petrocchi-Bartal & Khoza-Shangase, 2016), posing a threat to NHS (Kanji, 2018). The statistics from the HPCSA indicated that in January 2020, 788 audiologists and 1612 speech therapists and audiologists were registered (Khoza-Shangase & Kanji, 2021). Therefore, considering the shortage of audiologists documented within the African context, global access to human resources through tele-audiology for supervision needs to be explored (Khoza-Shangase & Kanji, 2021). Tele-audiology can then become another method of service delivery, and audiologists can serve as the programme managers with trained nurses and screeners conducting screening services (Khoza-Shangase & Kanji, 2021).

Limiting hearing screening to at-risk babies is not recommended (JCIH, 2007), but it may be a practical and feasible approach in resource-constrained contexts (Das et al., 2020). One should be cognisant that all programmes have to start somewhere, thus recognise that targeted or high-risk screening is a good foundation or starting point, compared to doing nothing at all, especially as the public sector is under-resourced (Kanji, 2018).

Amplification is seldom provided before 12 months to infants and children, which is far from the set standards recommended by the JCIH and HPCSA EHDI guidelines (HPCSA, 2018; JCIH, 2007; Khoza-Shangase et al., 2010). Thereafter, if the child has been fitted with hearing aids they may not be provided with appropriate and intensive aural rehabilitation as compared to the private sector because of the large workload in public. Research studies mainly focused on early detection and early intervention services are under-scrutinised (Störbeck & Young, 2016). Additionally, emphasis is greatly placed on NHS in the HPCSA guidelines however, one should be aware that the absence of diagnostic assessment implementation and intervention EHDI programmes cannot be successful (HPCSA, 2018). Research indicates that in South Africa there is a lack of a nationally agreed battery of protocols and tests to diagnose hearing loss in babies and infants (Khoza-Shangase & Kanji, 2021). Currently, there is no existing evidence-based method, which will decrease non-adherence towards hearing testing and intervention (Khoza-Shangase & Kanji, 2021).

### Facilitating and strengthening intersectoral collaboration

The third theme corresponded to knowledge and intersectoral collaboration between healthcare professionals and included the aspects related to knowledge, awareness, education, and training, and intersectoral collaboration. The feedback is summarised in Table 3.

**TABLE 3:** Strengths, weaknesses, opportunities, and threats for theme 3: Facilitating and strengthening intersectoral collaboration.

Variable	Subthemes: Awareness, knowledge, education and training
Strengths	Awareness of paediatricians and ENTs towards EHDI
Weaknesses	Lack of knowledge at a political level Poor knowledge levels among healthcare professionals Lack of collaboration between DoH and DoE Poor communication between audiologists in the private and public sectors
Opportunities	Mandate guidelines Creation of WhatsApp groups between professionals to improve collaboration and communication The involvement of nurses is a good way to catch babies at the clinics
Threats	Audiology services are viewed as non-essential Nurses have a lack of understanding and huge workloads

ENT, ear, nose and throat; DoH, Department of Health; DoE, Department of Education.

All of the participants stated that there is a general lack of awareness and knowledge about EHDI services among some healthcare professionals and within the different government sectors, which was identified as a weakness as stated in Table 3. Poor collaboration between audiologists in the public and private sectors was noted. The participants also reported little to no collaboration between the Department of Health and Education, rather a disconnect as indicated by the participants. A recommendation was the possible creation of WhatsApp groups between professionals working in the Department of Health and Education that could facilitate better service delivery:

‘[*B*]ecause what can happen is they can share policies, they can share information, expertise, and we can uplift the public side of our healthcare system. If we have a little bit more collaboration between … between both sides. I think that’s one thing I’d really like to see, which will facilitate screening services.’ (Participant F, female, bachelor’s degree, private sector)

Contrasting views were obtained from the participants regarding the inclusion of nurses for screening with some participants agreeing that nurses conducting screening when babies come for immunisations may assist with identification whereas others felt that nurses already have huge caseloads and are not knowledgeable about hearing loss. One opportunity identified was that if the HPCSA EHDI guidelines were made mandatory then institutions will be obliged to follow it and this will facilitate services.

The poor levels of awareness among healthcare professionals regarding EHDI and audiology services correlated with other study findings (Olusanya, 2008; Swanepoel, Ebrahim, Joseph, & Friedland, 2007; Swanepoel, Störbeck, & Friedland, 2009). Further correlating Ravi et al.’s study, that indicated NHS team members showed gaps in their knowledge, necessitating the need for educational and outreach programmes (Ravi, Gunjawate, Yerraguntla, & Rajashekhar, 2018). There have been no studies conducted in South Africa, to evaluate or describe the knowledge level and awareness of individuals working in government sectors and this should be an area that is explored. Audiologists need to advocate and promote EHDI and NHS, which can be done through private-public partnerships and non-government to government collaborations (Khoza-Shangase & Kanji, 2021). Communication and collaboration between professionals is a key aspect and effective EHDI services rely on a team approach (HPCSA, 2018). This will help facilitate a seamless flow of children from the health sector to the education sector.

Communication and collaboration are important as it can facilitate information sharing and enhance awareness about services offered, referral pathways could be developed to decrease the workload or waiting times, especially in public healthcare facilities. Current studies and guidelines recommend de-specialisation of hearing screening services to other personnel who will be trained and will have to adhere to regulated standards (Khoza-Shangase et al., 2017; Khoza-Shangase & Kanji, 2021). Equipment that is easy to use and sensitive, creates opportunities to use non-professionals as screeners, to reach contexts where audiologists are not available (Bezuidenhout, Khoza-Shangase, De Maayer, & Strehlau, 2021; Khoza-Shangase & Kanji, 2021).

There are successful programmes in the United Kingdom, which indicates 99% of parents allow for NHS services, which is conducted during a home visit by a nurse or in a hospital (WHO, 2010). Nurses being the backbone of primary healthcare, which is the first contact point in the healthcare system for more than 80% of South Africans, necessitates the need to ensure that this level is well resourced and equipped for implementation of NHS (Khoza-Shangase & Kanji, 2021). Most of the participants from Khan and Joseph’s (2020) study were agreeable towards nurses routinely screening for hearing loss during immunisation visits. Thereafter, once the programme is established further consideration should be given to better develop the programme in place (Khoza-Shangase & Kanji, 2021). The key successes and challenges of programmes should be shared by audiologists at forums to develop programmes at the national and provincial levels (Khoza-Shangase & Kanji, 2021). Regulations, ethics, and scopes of practice need to be adhered to if non-audiologists, like nurses, are involved in NHS (Khoza-Shangase & Kanji, 2021).

At a political level, mandating the guidelines would reduce challenges, thus individual preferences will not hinder best practice and facilitate healthcare professionals’ education and support (Scheepers, Swanepoel, & Le Roux, 2014). According to the study by Khan and Joseph (2020), practitioners had a positive view towards hearing screening being mandatory and that it should form part of the birth package offered to mothers. Currently, UNHS has not been mandated by the Department of Health and additionally, there is a scarcity of contextually relevant evidence-based challenges regarding the implementation of NHS in the public healthcare sector (Bezuidenhout et al., 2018). Hearing screening programme performance needs to be audited through formalised evaluations of pilot programmes, which should include primary, secondary and tertiary healthcare contexts which are coordinated by the Department of Health in conjunction with tertiary hospitals (HPCSA, 2007, 2018).

### Evaluating protocols and procedures for screening and managing follow-up

The fourth theme related to evaluating the process and methods for screening and thereafter the follow-up, and included aspects linked to screening platforms and procedures and the follow-up rates. The feedback is summarised in Table 4.

**TABLE 4:** Strengths, weaknesses, opportunities, and threats for theme 4: Evaluating protocols and procedures for screening and managing follow-up.

Variable	Subthemes: Follow-up rates, protocols, and procedure
Strengths	Initial contact to build and foster relationships with parents Wider application of AABR as it is not dependent on middle ear state ABRs in private conducted in the theatre
Weaknesses	New mothers are often overwhelmed and may forget about the test Reliability and validity of OAE testing in hospitals AABR is invasive and time-consuming Sedation prescribed for electrophysiological testing
Opportunities	Inclusion of AABR if baby fails an OAE Ensuring mothers are aware that a pass on an OAE does not mean the child will not have a hearing problem in the future Collaboration between doctors and audiologists to develop a good sedation protocol Inclusion of open days at hospitals
Threats	High rate of loss-to-refer or poor follow-up Medical aids are not paying for hearing screening in private OAE highly dependent on middle ear state

AABR, automated auditory brainstem response; OAE, otoacoustic emissions; ABR, auditory brainstem response.

A threat identified by the participants at an institutional level is the high rate of loss-to-refer or the poor follow-up rate, experienced in both the public and private healthcare settings. Another threat as noted in Table 4, indicated that the medical aids, specifically for the private sector, are not paying for the hearing screening. Some participants mentioned that using otoacoustic emissions (OAEs) to screen babies before discharge from the hospital may not be a practical method, as it is highly dependent on the middle ear state, size of the baby’s ear, and state of the baby. The inclusion of an automated auditory brainstem response (AABR) test was noted to be debatable between A/STAs as strengths and weaknesses were identified as stated in Table 4:

‘I’m always worried about giving the results to mom if the baby has passed the OAE because we don’t know about those auditory neuropathies … so I feel that we are missing that … but that is my personal opinion … I always maintain that.’ (Participant E, female, bachelor’s degree, private sector)

In public healthcare facilities, the sedation provided to the child, specifically for electrophysiological testing, was reported to be problematic, as in many instances the infant or child does not fall asleep and they are rebooked to redo or continue the test. An opportunity mentioned was the collaboration between doctors and audiologists in developing a good sedation protocol for all health professionals to follow, specifically for babies requiring further electrophysiological testing.

The EHDI guideline states that screening should be conducted before discharge (HPCSA, 2018). It can be problematic as babies’ ears can have vernix, birth fluid, or wax, which can prevent obtaining a true result. According to Khoza-Shangase and Kanji (2021), hearing screening is possible postpartum within 6 hours. However, it is more practical and efficient to screen infants at their 3-day appointment after birth, which can significantly reduce false positives. Screening using AABR provides advantages of a higher rate of true positives, lower referral rates, effective screening at a younger age, and the ability to identify neural hearing losses (De Kock, Swanepoel, & Hall, 2016). It should be noted that there have been new developments in AABR technology, which addresses the problems with preparation, test time, and disposable costs (Cebulla & Shehata-Dieler, 2012; Cebulla, Hofmann, & Shehata-Dieler, 2014) and are broadening the application opportunities for AABR screening even in community-based contexts settings (De Kock et al., 2016).

Auditory brainstem response (ABR) testing under sedation is currently the gold standard and is used to diagnose hearing loss in young children and infants who are unable to complete behavioural testing or are not developmentally ready (Abulebda et. al., 2017). There is no specific sedation protocol that is recommended by the HPCSA EHDI guideline (HPCSA, 2018). Therefore, hospitals follow different protocols, depending on what is prescribed by general practitioners or doctors. The type of sedation prescribed influences whether the infant or child will sleep long enough for the necessary audiological testing. Sometimes the child may not sleep, affecting the reliability of the test results, causing the child to be rebooked, further causing delays in diagnosis and intervention.

The loss to follow-up or loss-to-refer is a challenge in NHS programmes worldwide (HPCSA, 2018). Reducing the poor follow-up rates necessitates the need for proactive reminders and more effective communication with caregivers (Scheepers et al., 2014). The outpatient follow-up rescreen should be available to families, without barriers like language, literacy levels, transportation, or cost (Thomson & Yoshinaga-Itano, 2018).

### Engaging, understanding, and supporting caregivers or families

The fifth theme alluded to caregiver and family factors and included aspects related to socio-economic factors, traditional and cultural factors, and knowledge of parents and caregivers. The feedback is summarised in Table 5.

**TABLE 5:** Strengths, weaknesses, opportunities, and threats for theme 5: Engaging, understanding, and supporting caregivers or families.

Variable	Subthemes: socio-economic factors, traditional and cultural factors, knowledge of parents/ caregivers
Strengths	More outreach services Empowering parents with knowledge for decision making
Weaknesses	Cost for test in urban areas Cost for travelling in rural areas Limited understanding and poor knowledge levels in rural areas Cultural reasons affecting follow-up appointments
Opportunities	Importance of counselling parents or caregivers and families More aware of cultural and traditional beliefs and views Education and awareness promotion Creation of pamphlets and posters
Threats	Lack of availability and access to services in rural areas Urban versus rural disparity The cycle of grief experienced by parents or caregivers needs to be addressed

The barriers, as reported by the participants, included the cost of transport for parents or caregivers especially in the rural areas, and the cost of the test in private healthcare. In rural areas, there is a lack of availability of audiology services which results in patients having to travel far to get access to services that they require. Results from the participants revealed some traditional and cultural beliefs posed as a challenge, but those working in rural areas reported it to be a barrier impacting follow-up rates:

‘So I gave her a follow-up date for four weeks and she told me I can’t make it when the baby is four weeks because culturally, she’s not allowed to leave her house once she’s discharged from hospital. So I think culture can affect … definitely can affect …’ (Participant F, female, bachelor’s degree, private sector)

The participants indicated that parents may be more knowledgeable about audiology and EHDI services in private compared to public healthcare facilities, as A/STAs may be more visible. A threat to EHDI as reported by the participants was the cycle of grief experienced by parents or caregivers, and opportunities to aid in this have been identified in Table 5.

Logistical barriers to EHDI as reported by the participants correlated with the results from Khoza-Shangases’s (2019) study. Studies have indicated that there are significantly greater barriers related to access, experienced by rural versus urban communities, which include time, distance, and cost of receiving healthcare services (Jackson et al., 2006; Stuckler, Basu, & McKee, 2011). There is a need for reliable and affordable transport, especially because of the large distances and limited healthcare facilities in rural communities (Gaede & Versteeg, 2011). Service delivery and access need to be driven depending on the needs of individuals being served (Khoza-Shangase, 2019).

South Africa has a richly diverse population in culture and language, thus clinical interactions need to be conducted using a mode or language of communication which patients understand (HPCSA, 2019). However, English is a dominant language used for communication and imparting health knowledge orally or through handouts in public healthcare (Janse van Rensburg, 2020). This education is essential for the prevention of disease and promotion of health, but many individuals may have poor ability to understand what they read or hear (Griffen, Mckenna, & Tooth, 2006). Therefore, culturally and linguistically appropriate management should be provided by affording unbiased and fair opportunities to people from different cultural and language groups seeking services (HPCSA, 2019). Professionals should be knowledgeable about various cultural practices that can influence care and reflect on personal discomforts, such as cultural biases which will aid in providing family-centred care (Grandpierre et al., 2019).

Traditional healing also plays an important role in South African’s health-seeking behaviours, with traditional healers being synonymous in black African communities (Pillay & Serooe, 2019). Eight out of 10 black people from South Africa are believed to use only traditional health practitioners or in conjunction with Western medicine (Ross, 2010), other studies indicated approximately 70% of black South Africans use traditional health practitioners (Bopape, 2016; Latif, 2010; Ramgoon, Dalasile, Paruk, & Patel, 2011). In South Africa, traditional healing methods are highly debatable because of the controversies surrounding the possible negative impacts of traditional healing (Pillay & Serooe, 2019).

Caregivers and parents need to be part of the assessment process which will aid in their understanding of the assessment management process (Kovacs, 2012). Attention should be provided to create awareness and counselling should include the entire family, as an individual member’s poor knowledge can lead to a delay in identification and management of the hearing loss (Rajagopalan et al., 2014). Data indicates that the resolution of grief with early identified children and their families may occur faster compared to later-identified children, but only if these children develop strong communication and language skills (Yoshinaga-Itano, 2003). South Africa has very little data regarding caregiver perceptions of early identification of hearing loss (HPCSA, 2018) and further research should be conducted towards ‘culturally congruent screening programmes’ (HPCSA, 2018, p. 23).

## Conclusion

Research indicates that early intervention principles were not applicable for South Africa, because of language barriers, socio-economic factors, cultural diversity, lack of resources, and awareness which affect audiology service delivery (Khoza-Shangase et al., 2010). The main barriers affecting EHDI implementation included lack of resources, poor follow-up rates, limited knowledge and education, socio-economic status, and practicality of the EHDI guidelines. These findings emphasise the need for context-specific solutions and strategies to facilitate effective practice and implementation of EHDI services, because of the rich and diverse contexts:

Children with hearing loss are as much part of the future of the country as those with normal hearing and it is through effective EHDI services that the active and equal participation of these children will be secured among their hearing peers to change, influence and direct the future of South Africa. (HPCSA, 2018, p. 47)

### Limitations and recommendations

The study sample and size are representative of A/STAs employed in private and public healthcare facilities in the KZN province only and therefore, the results may not be generalisable in a probabilistic sense (Marshall & Rossman, 2010). The time limit of 45 min – 60 min scheduled for the telephonic interview may have affected the depth of information obtained from the participants, as many A/STAs working in healthcare facilities have very busy schedules. Audio recordings of the telephonic interviews had certain limitations as the quality of recording of one or two of the interviews was affected because of the background noise, in the participant’s workplace (Howitt, 2016). Therefore, certain words/phrases in the audio recordings were unable to be transcribed.

It may be necessary for de-specialisation of hearing screening services to healthcare professionals such as nurses to facilitate UNHS and achieve goals and principles as set out by the HPCSA EHDI (HPCSA 2018) guideline. Future research should investigate the barriers and facilitators of EHDI implementation, in the various provinces, using a larger quantitative, online survey-based study in South Africa. Assessment and evaluation of resources and protocols available in healthcare facilities in all the provinces of South Africa should be conducted, which can assist with developing proper EHDI programmes and for implementation purposes. A national database can assist in obtaining accurate prevalence rates for newborn and infant hearing loss, in the South African context and provide information regarding the age of hearing screening and diagnosis of hearing impairment (HPCSA, 2018).

## Appendix 1

### Semi-structured telephonic interview schedule

The broad SWOT Framework was used to aid in guiding the telephonic interview questions. The participants had to elaborate on some or all of the levels in relation to the strengths, weaknesses, opportunities and threats.

**Table:**

Strengths	What are the various factors that contribute towards EHDI success in South Africa in places where it is implemented?		Professional level Political level Institutional level Educational level Practice level Social level
Weaknesses	What are the challenges and barriers to successful EHDI implementation in South Africa?		
Opportunities	What are some strategies/trends/guides that can be used in South Africa to improve EHDI service delivery?		
Threats	What are the obstacles and threats faced in South Africa, affecting EHDI implementation and practice?		

In addition, the following are ***examples*** of trigger questions that were used to guide the telephonic interview process.			
The EHDI guideline was released by the HPCSA, first in 2007, and a revised version in 2018. In your view, how have the guidelines enabled progressive and successful EHDI implementation in South Africa? What are the barriers and challenges experienced at each of the levels (professional, political, institutional and so forth) in implementing the EHDI guidelines (2007, 2018) as envisaged? EHDI benchmarks state that all infants should have their initial screening by no later than 1-month and 6-weeks for infants at clinic based programmes. What are your thoughts regarding the practicality of screening at 1-month and at 6-weeks for infants at clinic based programmes? Are you involved in any UNHS/NHS programme or are aware of any such programmes? What protocols are followed? Do they vary considerably from hospital to hospital? In the absence of UNHS certain institutions conduct targeted or risk-based screening and others screen on referral based systems. What has been your experience in the context/s you work in? According to the EHDI guideline infants should be diagnosed with a hearing loss by 3-months, no later than 4-months. Intervention should be provided by no later than 6-months and 8-months for those in clinic-based settings. What are your thoughts regarding these benchmarks? Return rates for rescreens and/or diagnosis following the initial NHS or infant screening generally is problematic globally. What in your opinion are the barriers (threats and weaknesses) at the different levels (political, professional etc.) in South Africa affecting follow up or return rates? Data management is an essential part in screening to track patients, monitor and evaluate the effectiveness of a UNHS/NHS or EHDI programme overall. What has been your experience with data management?			

EHDI, early hearing detection and intervention; UNHS, universal newborn hearing screening; NHS, newborn hearing screening; HPCSA, Health Professions Council of South Africa; SWOT, strengths, weaknesses, opportunities, threats.
